# From Punishment to Treatment: The “Clinical Alternative to Punitive Segregation” (CAPS) Program in New York City Jails

**DOI:** 10.3390/ijerph13020182

**Published:** 2016-02-02

**Authors:** Sarah Glowa-Kollisch, Fatos Kaba, Anthony Waters, Y. Jude Leung, Elizabeth Ford, Homer Venters

**Affiliations:** Correctional Health Services, New York City Health + Hospitals, New York, NY 11101, USA; sglowakollisch@nychhc.org (S.G.-K.); fkaba@nychhc.org (F.K.); awaters1@nychhc.org (A.W.); yleung2@nychhc.org (Y.J.L.); eford@nychhc.org (E.F.)

**Keywords:** jail, solitary confinement, mental health, self-harm

## Abstract

The proliferation of jails and prisons as places of institutionalization for persons with serious mental illness (SMI) has resulted in many of these patients receiving jail-based punishments, including solitary confinement. Starting in 2013, the New York City (NYC) jail system developed a new treatment unit for persons with SMI who were judged to have violated jail rules (and previously would have been punished with solitary confinement) called the Clinical Alternative to Punitive Segregation (CAPS) unit. CAPS is designed to offer a full range of therapeutic activities and interventions for these patients, including individual and group therapy, art therapy, medication counseling and community meetings. Each CAPS unit requires approximately $1.5 million more investment per year, largely in additional staff as compared to existing mental health units, and can house approximately 30 patients. Patients with less serious mental illness who received infractions were housed on units that combined solitary confinement with some clinical programming, called Restrictive Housing Units (RHU). Between 1 December 2013 and 31 March 2015, a total of 195 and 1433 patients passed through the CAPS and RHU units, respectively. A small cohort of patients experienced both CAPS and RHU (*n* = 90). For these patients, their rates of self-harm and injury were significantly lower while on the CAPS unit than when on the RHU units. Improvements in clinical outcomes are possible for incarcerated patients with mental illness with investment in new alternatives to solitary confinement. We have started to adapt the CAPS approach to existing mental health units as a means to promote better clinical outcomes and also help prevent jail-based infractions. The cost of these programs and the dramatic differences in length of stay for patients who earn these jail-based infractions highlight the need for alternatives to incarceration, some of which have recently been announced in NYC.

## 1. Introduction

The United States has the highest rate of incarceration in the world, with approximately 11.6 million people cycling through jails (in New York State: locally operated, short-term facilities that hold people awaiting trial and/or sentencing, as well as those found guilty and sentenced to a term of one year or less) and prisons (long-term facilities run by a State or the Federal government that hold people serving sentences of longer than one year) annually [1]. Approximately 95% of these incarcerations occur in jails, which are chaotic settings given the short stay of the incarcerated and the lack of established programs that exist in most prisons [2,3]. The New York City (NYC) jail system is the nation’s second largest, with 70,000 annual admissions and 11,000 persons incarcerated at any given time. The median length of incarceration is 8 days. In the NYC jail system, the Bureau of Correctional Health Services (CHS) of the NYC Department of Health and Mental Hygiene (DOHMH) is responsible for all aspects of health care for the incarcerated, while the NYC Department of Correction (DOC) is responsible for security. Approximately 25% of those admitted to the jails will become part of the mental health service, of whom only a small percentage will ultimately be designated as having serious mental illness (SMI). Although the proportion of SMI patients has remained relatively stable in recent years, the percentage of persons who are followed by the mental health service has increased from approximately 12% in 2004 to 25% today. Because persons with mental illness have a 50% longer length of stay than others, on average, they now represent approximately 38% of persons in jail at any given time, despite only 25% of any admission cohort becoming a mental health patient.

Health providers in jails and prisons care for patients with high rates of substance use, mental health and chronic medical concerns [4,5]. In addition, these patients experience morbidity and mortality specifically related to their incarceration, ranging from medication interruption to injury to worsening mental health during solitary confinement, and mortality. Despite widespread use in American jails and prisons, solitary confinement is a practice that has been associated with adverse health consequences to those placed in these settings and unclear benefits to security or safety [6,7]. Solitary confinement involves being placed alone in a cell for 22–24 h per day as punishment for violating correctional rules or as a preventive measure because someone has been deemed too dangerous to be around other inmates. These types of solitary confinement are often termed punitive and administrative segregation, respectively, and they are considerably more expensive than other management approaches because of their requirement for additional correctional officer and health services staffing. Internal reviews conducted by CHS have revealed that these types of units are also the site of disproportionate levels of violence for both staff and inmates.

In 2012, CHS adopted a human rights framework to the NYC jail health mission, including an examination of concerns associated with solitary confinement [8]. An early quality improvement project of our human rights subcommittee was to analyze health outcomes associated with self-harm among patients in jail [9]. This analysis of approximately 225,000 jail admissions between 1 January 2010 and 31 October 2012 revealed that predictors of self-harm included SMI, ever being placed in solitary confinement and being an adolescent. Although only 7.3% of patients in this study ever passed through solitary confinement, they represented over half (53.3%) of all self-harm and almost half (45.0%) of high-lethality self-harm. Based on these data and concerns from advocates and other policy makers, DOC and CHS designed and implemented an alternative to solitary confinement for persons with serious mental illness who received infractions for violating jail rules. This unit came to be called the Clinical Alternative to Punitive Segregation (CAPS) unit. While the CAPS unit was designed to replace solitary confinement for patients with SMI, those with lower levels of mental illness would continue to experience solitary confinement, in units called Restrictive Housing Unit (RHU). The RHU aims to combine solitary confinement with some clinical interventions via an incentive-based system. At the most advanced level, patients in the RHU can earn up to 4 h out-of-cell time each day, leaving the bulk of their experience as solitary confinement. Prior to this initiative, those patients who violated jail rules and sentenced to solitary confinement, but were found to be too mentally ill to sustain that placement were primarily directed to the Mental Health Assessment Unit for Infracted Inmates (MHAUII), which housed 200 patients at the time of closure, approximately 25% of whom met criteria for SMI. Very few clinical services were available to these patients, aside from cursory cell-side rounds and occasional out of cell encounters in the jail health clinics. When the decision was made to close MHAUII and the first men’s CAPS program opened in November 2013, patients were transferred into either CAPS or the RHU.

The CAPS units were designed as clinical settings where patients would not be locked in isolation, but would instead participate in a comprehensive schedule of therapeutic activities, including psychotherapy, creative art, nursing education groups, individual mental health and medical encounters and community meetings with patients, health and security staff (Table 1). The CAPS units are lock-out units, meaning patients are encouraged to spend their days outside their cells interacting with others unless there is a clinical reason to be in their cell. Patients in the NYC jail system are categorized by their level of mental health needs. Any patient who has three or more encounters with the jail mental health service is designated as “M Status”, while those patients designated as having SMI are those with more serious needs and a higher level of functional impairment, based on criteria established by the NY State Office of Mental Health [10]. In general, the patients identified for CAPS placement were those with SMI.

**Table 1:** CAPS Overview.

Time/Activity	Description/Staff
7:00 Count	DOC; officer will count all patients.
7:30 Security Inspection	DOC; officer will conduct a security inspection.
8:00–8:30 Staff Communication Meeting	All clinical staff and DOC officers; discuss issues from the previous tours and to set the tone for the day.
9:00–9:30 Rounds	Mental Health Treatment Aides will round on all patients on Suicide Watch, Mental Health Clinicians and Psychiatrists will round together on the entire unit’s patient census.
9:30–9:45 Community Meeting	All clinical and DOC staff with all patients; set the tone for the day. New patients will be introduced to the treatment milieu.
9:45–11:00 Clinical Group Programming	Mental Health Clinician, Mental Health Treatment Aides supplemented with Clinical Supervisors and Therapists. DOC officers will remain on the periphery during clinical programming sessions unless needed to respond to an emergency.
11:00–11:30 Lunch	DOC with the assistance of the Mental Health Treatment Aides.
12:00–13:00 Therapeutic Activity Group	Therapists (art, music, yoga, writing) with treatment aides; therapeutic groups supplement the clinical groups and provide an opportunity for the patients to engage in art, music, yoga and writing exercises.
13:00–14:00 Clinical Group Programming	Mental Health Clinician, Mental Health Treatment Aides supplemented with Clinical Supervisors and Therapists. DOC officers will remain on the periphery during clinical programming sessions unless needed to respond to an emergency.
14:30–16:00 DOC Count/Treatment Team Case Conference	DOC: officers will count the patients and conduct a security inspection. Mental Health Treatment Aides; available to interact with patients as necessary to ensure compliance, however this time will allow patients a break from programming. During this time the clinical treatment team will meet (Clinical Supervisor, Mental Health Clinician, Psychiatrist and Mental Health Treatment Aide) to discuss the patient population, address treatment issues.
16:00–17:00 Rest/Individual Treatment	Mental Health Treatment Aides will remain on the unit to perform their duties. Patients are encouraged to rest during this down time and to complete any homework assignments they may have. Individual treatment will take place during this time on the unit by (Mental Health Clinicians and Psychiatrists).
17:00–17:30 Dinner	DOC officers with the assistance of Mental Health Treatment Aides will be responsible for overseeing the provision of all meals on the unit. They will ensure that all patients are fed.
17:30–18:00 DOC and Clinical Staff Communication Meeting	All clinical and DOC staff will be in attendance to discuss issues from the previous tours and to set the tone for the evening programming.
18:00–19:00 Clinical Group Programming	Mental Health Clinician, Mental Health Treatment Aides supplemented with Clinical Supervisors and Therapists. DOC officers will remain on the periphery during clinical programming sessions unless needed to respond to an emergency.
19:00–20:00 Rest/Individual Treatment	Mental Health Treatment Aides will remain on the unit to perform their duties. Patients are encouraged to rest during this down time and to complete any homework assignments they may have. Individual treatment will take place during this time on the unit by (Mental Health Clinicians and Psychiatrists).

A key design component of the CAPS unit was to form a team with health and security staff working together to promote improved clinical and security outcomes. The CAPS units typically employ dedicated social workers (4), psychologist (1), nurse (1), psychiatrist (0.5) and mental health treatment aides (4) located on each unit, which is a far richer staffing matrix than that of traditional mental health units. The inclusion of mental health treatment aides in the CAPS units is new for our jail system. These staff are responsible for assisting patients in groups and other therapeutic activities on the unit, documenting and communicating therapeutic observations to other staff, and engaging with patients throughout the day who may be experiencing difficulty. Also new to our jail system is having a comprehensive mental health staff based within the unit’s housing area, with a dedicated office space inside the unit and narrow focus on the relatively small population housed therein. Prior to CAPS opening, both security and health staff who were identified as wanting to work on these units participated in a week of joint training, with emphasis on de-escalation and communication.

After one year of operation, we conducted a program evaluation to review health outcomes in the CAPS units that we had earlier reported on: self-harm and injury. Because of the different pathways for entry to CAPS and RHU, we anticipated that the patient profiles in these settings would differ significantly, with fewer SMI patients in RHU. As DOHMH and DOC enact further limitations on solitary confinement and also introduce new initiatives to reduce jail-based violence, the CAPS unit will serve as a model for other settings where patient engagement can be improved.

## 2. Methods

Using our electronic health records, we created three cohorts of people who were incarcerated between December 2013 and March 2015, committed infractions of jail rules and, instead of being sent to standard solitary confinement, were sent to CAPS, sent to the RHU, or sometimes sent to RHU and sometimes sent to CAPS. A subset of the CAPS cohort had also recently spent time in MHAUII, and this group was used a comparison to those who spent time in both CAPS and RHU, but it was not mutually exclusive to the primary three cohorts, so was not used in our primary analysis. For those patients whose first CAPS or RHU placement during the study period was between January and March 2015, we tracked outcomes through June 2015 to provide enough time for observation of results.

For each of these cohorts, we conducted descriptive analyses and looked at demographic data, including age, sex, mental health status, and length of stay—both in jail and within the examined program cohorts. When calculating lengths of stay and corresponding person-days, for those people still incarcerated and/or still enrolled in either CAPS or RHU at the end of the study period, the last day of the study period was used as the end date for incarceration and/or program enrollment. We examined patients who were both admitted and discharged during the study period as well as those who were either admitted or discharged during the study period. Given the demographic discrepancies for the CAPS and RHU cohorts, we did not conduct statistical analysis to compare those two cohorts, instead only doing so for those who spent time in CAPS and those who spent time in both CAPS and RHU. Each line of our data corresponded to an incarceration, which means that each cohort may have contained multiple instances of a single person who had multiple independent incarcerations during the study period.

For clinical outcomes, we focused on incidents of self-harm and verified injuries. We have previously identified both solitary confinement and serious mental illness as risk factors for self-harm and one of the goals of this analysis was to identify whether the CAPS unit is associated with lower rates of self-harm. Acts of self-harm are defined as intentional injuries to self, and these gestures are described here in terms of frequency and the cause or method of injury (e.g., lacerations, banging head, and hanging). Injury encounters occur when patients are seen in the jail health clinic for a self-reported injury or from mandated encounters such as experiencing a use of force with security staff or being observed in an altercation with another patient. For encounters where there is physical evidence of injury, the injury is classified as “verified”.

Our first stage of statistical analysis was an effort to understand program effectiveness by comparing the verified injury and self-harm rates per patient between time spent in CAPS and time spent out of CAPS for the patients who were only enrolled in the CAPS program (Analysis 1). Our second stage of statistical analysis was in comparing the rates of verified injuries and self-harm per patient for those patients who spent time in both CAPS and RHU (Analysis 2).

We used univariate negative binomial regression in order to compare sums of verified injuries and self-harm between in CAPS and out of CAPS for the CAPS only cohort, for over-dispersed count outcome data. We utilized the same method to compare the differences between in-CAPS, in-RHU and neither CAPS nor RHU verified injuries and self-harm for the CAPS/RHU cohort.

Data extrapolation and mining were conducted using Excel 2010 (Microsoft Inc., Redmond, WA, USA). Descriptive and statistical analyses were conducted using SPSS PASW Statistics 23, Release Version 23.0.0 (IBM Corp., Armonk, NY, USA) and SAS, Version 9.2 (SAS Institute, Inc., Cary, NC, USA). All of the methods referenced in this report constitute quality improvement and public health surveillance and were exempt from Institutional Review Board review.

## 3. Results

### Descriptive

The cohorts of those patients who spent time in the CAPS program, those in the RHU program, and those who were housed in both CAPS and RHU during the study period were not the same size, and had disparate demographic makeups (Table 2). There were 195 in the CAPS-only cohort, 1433 in the RHU-only cohort, and 90 in the CAPS/RHU cohort. Age distributions were roughly similar for all three cohorts, with the exception that more adolescents (age 16–18) were in the RHU. This reflects the less common designation of adolescents as SMI. Sex distribution was also similar across the cohorts, with the exception of there being fewer female patients in RHU. Nearly all included patients had an M designation, but a majority of patients in the CAPS (87.2%) and CAPS/RHU (74.4%) cohorts had SMI, in comparison to RHU (21.7%). CAPS-enrolled patients spent an average of 70 days in the program, with an average of 255 days in jail, while those in RHU spent an average of 46 days in the program and an average of 367 days in jail, and those in both programs spent an average of 46 days in CAPS and 52 days in RHU and 383 days in jail. While people were not sentenced to particular lengths of time in CAPS, as they were in RHU and MHAUII, but instead stay in the program until mental health staff determine they should be returned to their a less intensive treatment unit, they did not seem to graduate out of the program as initially envisioned. As there was not a sufficiently therapeutic setting to return them to, it seemed the mental health staff frequently kept patients in CAPS until they were released from jail.

**Table 2:** Demographics.

	CAPS Only	RHU Only	Both CAPS and RHU	
Cohort	195	1433	90	
Age				
Mean, in years	33	27	30	
18 and under	5 (2.6%)	257 (17.9%)	2 (2.2%)	
19 and over	190 (97.4%)	1176 (82.1%)	88 (97.8%)	
Sex				
Male	133 (68.0%)	1307 (91.2%)	59 (65.9%)	
Female	62 (32.0%)	126 (8.8%)	31 (34.1%)	
Mental Health Status				
M status (in MH service)	195 (100.0%)	1403 (97.9%)	90 (100.0%)	
SMI status (subset of M)	170 (87.2%)	311 (21.7%)	67 (74.4%)	
Length of Stay				
Total jail LOS (mean, per person, in days)	255	367	383	
Program LOS (mean, per person, in days)	70	46	46 (CAPS)	52 (RHU)

There were 26 patients who were in both CAPS and MHAUII (16 of whom were also in RHU), and we didn’t conduct statistical analysis due to the small sample size. However, we did conduct a crude analysis, finding higher rates of both verified injury and self-harm. A higher percentage of people in both CAPS and MHAUII (100%) had verified injuries than those in both CAPS and RHU (75.8%). The rate of injury (described as incidence per 100 person-days) was nearly double when in MHAUII (0.94) then when in CAPS (0.54). Not as high a proportion of those in CAPS and MHAUII (26.9%) engaged in self-harm, compared to those in CAPS and RHU (34%). For those in CAPS and MHAUII, the rate of self-harm (described as incidence per 100 person-days) when in MHAUII (0.38) was over six times higher than when in CAPS (0.06).

#### Analysis 1: Comparing Clinical Outcomes of the CAPS Cohort—While in CAPS and While Out of CAPS

Approximately a quarter of those in CAPS (24.2%) engaged in self-harm, while one-third (33.3%) of those in both CAPS and RHU did (Table 3). The highest rate of self-harm (described as incidence per 100 person-days) was found for those spending time in both CAPS and RHU: their rate was nearly five times higher when in RHU (0.98) than when in CAPS (0.19). Following that, the next highest rate was for those in the CAPS cohort, and the rate of incidence of self-harm was approximately the same while in CAPS (0.47) and out of CAPS (0.48). Rate ratio of self-harm incurred in CAPS with reference to out of CAPS is 0.6829 (95% CI 0.3159–1.4763) and not statistically significant (*p* = 0.33).

**Table 3:** Self-Harm.

	CAPS		Both CAPS and RHU		
	In CAPS	Out of CAPS	CAPS	RHU	Non-CAPS Non-RHU
Total cohort	195		90		
Total length of stay (in person-days)	14,490	36,043	4156	4687	25,642
Total self-harming patients	48 (24.2% of total)		30 (33.3% of total)		
Men	46 (34.5% of all men)		24 (41.0% of all men)		
Women	2 (3.2% of all women)		6 (19.4% of all women)		
18 and under	2 (40.0% of all ≤18)		2 (100.0% of all ≤18)		
19 and over	46 (24.2% of all ≥19)		28 (32.0% of all ≥19)		
Patients with:					
1 act of self-harm	17 (35.4%)		16 (53.3%)		
2–3 acts of self-harm	10 (20.8%)		9 (30.0%)		
4 or more acts of self-harm	21 (43.8%)		5 (16.7%)		
Range of acts of self-harm/patient	1–37		1–27		
	1–6	1–37	1–6	1–15	1–27
Total acts of self-harm	262		190		
	68	172	8	46	136
Rate of incidence of self-harm (per 100 person-days)	0.47	0.48	0.19	0.98	0.53
Type of self-harm:					
Lacerations and/or scratches	140 (53.4%)		86 (45.2%)		
Banging head or other body part	29 (11.1%)		25 (13.2%)		
Hang-up or attempted	18 (6.9%)		14 (7.4%)		
Swallowing something (e.g., batteries, soap)	41 (15.6%)		32 (16.8%)		
Other *	34 (13.0%)		33 (17.4%)		

* Other acts of self-harm include those related to: tying a sheet around the neck, overdosing on medication, setting cell or self on fire, and combinations of other acts.

#### Analysis 2: Comparing Clinical Outcomes of the CAPS/RHU Cohort—While In CAPS, While in RHU, and While in Neither CAPS nor RHU

In looking at the cohort of people who spent time in both CAPS and RHU, we compared the rate of verified injuries and acts of self-harm while housed in CAPS, while housed in RHU, and while housed in other settings. We found that the rate ratio for acts of self-harm per patient, controlling for length of stay in each setting, was 2.24 for RHU (95% CI, 0.8201–6.162, *p* = 0.12) and 2.34 for other settings (95% CI, 0.8559–6.1416, *p* = 0.098), but was not statistically significant for either. We found that the rate ratio for verified injuries per patient, controlling for length of stay in each setting, was 2.23 for RHU and 1.86 for other settings (Table 4). This indicates that the rate of verified injuries per patient while in CAPS (0.69) was lower than in any other setting, though it was only statistically significant in comparison to the rate of verified injuries per patient while in RHU (95% CI, 1.1278–4.4155, *p* = 0.021), and not statistically significant in comparison to other housing settings (95% CI, 0.9354–3.689, *p* = 0.076).

**Table 4:** Verified Injury.

	CAPS		Both CAPS and RHU		
	In CAPS	Out of CAPS	CAPS	RHU	Non-CAPS Non-RHU
Total cohort	195		90		
Total length of stay (in person-days)	14,490	36,043	4156	4687	25,642
Total patients with verified injuries	104 (53.3% of total)		67 (74.7% of total)		
Men	89 (66.7% of all men)		46 (78.3% of all men)		
Women	16 (25.8% of all women)		21 (67.7% of all women)		
18 and under	3 (60% of all ≤18)		2 (100% of all ≤18)		
19 and over	102 (53.4% of all ≥19)		65 (74.2% of all ≥19)		
Patients with:					
1 verified injury	38 (36.5%)		22 (32.8%)		
2–3 verified injuries	31 (29.8%)		21 (31.3%)		
4 or more verified injuries	35 (33.7%)		24 (35.8%)		
Range of verified injury/patient	1–40		1–48		
	1–26	1–36	1–3	1–19	1–48
Total verified injuries	428		337		
	132	296	25	72	238
Rate of incidence of verified injury (per 100 person-days)	0.91	0.82	0.69	1.58	0.93

The cohort containing the highest proportion of patients with verified injuries was for those spending time in both CAPS and RHU (74.7%), followed by the CAPS cohort (53.3%). The rate of injury (described as incidence per 100 person-days) was highest within the CAPS/RHU cohort, with their rate higher when in RHU (1.58) than when in CAPS (0.69). The rate ratio of verified injuries incurred in CAPS *vs.* out-of-CAPS (reference) is 1.13 (95% CI, 0.6914–1.8478) and not statistically significant (*p* = 0.63).

## 4. Discussion

This analysis contains several important limitations. First, the small sample size of these cohorts limits the ability to detect meaningful differences in outcomes. Second, all analyses were conducted on the basis of individual incarcerations, which means that some people appeared multiple times within cohorts. Third, the focus on setting and treatment may be confounded by other baseline differences (e.g., type of diagnosis, prior incarceration). As we adapt the CAPS model to other mental health units, we will accrue large numbers of patients for similar analysis, and include other important outcomes, such as medication adherence and hospitalization. More broadly, the primary goal of this analysis was to report progress on an alternative to solitary confinement for persons with mental illness in a jail setting. The need to provide such alternatives inside jail is not in question, and both legal and clinical authorities have created a mandate for this approach in multiple states across the United States. As more correctional settings develop alternatives to solitary confinement, there will be an important opportunity for more rigorous analysis of the costs and benefits associated with treatment approach.

These data support the objective of the CAPS unit; to provide a therapeutic alternative to solitary confinement for patients with SMI. For jail administrators and correctional health systems, there is a pressing need for approaches that can meet the clinical needs of patients, while also improving security within the setting. Because of the baseline differences between the CAPS and RHU cohorts of patients, the most meaningful comparison is among those patients who experienced both settings. Within this cohort, significantly lower rates of self-harm and injury occurred during treatment in the CAPS unit as opposed to punishment in the RHU. These clinical outcomes are important for the health of individuals, but also for the jail system. Every injury or act of self-harm requires significant clinical and security responses. In addition, when injuries stem from use of force, correctional staff come under scrutiny. Finally, these clinical outcomes may also bear on the ability to produce patients for their court appearances.

Persons who were on the CAPS and RHU units experienced 2–3 times longer jail stays than those who remained off these units but who had similar levels of mental health concerns. This observation is further complicated by the long amount of time they spent in jail before their transfer to CAPS. One possible explanation is that the rules and stress of the jail system may pose increasing challenges to these patients, resulting in higher rates of infraction and behavioral problems as time passes. How these difficulties in jail relate to length of stay is unclear; however, these patients may face greater hurdles in securing bail, and also in participating in their criminal defense.

Based on the success of the CAPS model, we secured funding to adapt this approach to several other mental health units. We have approximately 30 mental health units throughout the jails, and patients are transferred to these settings when they require higher levels of monitoring and care. Despite having more resources than general population housing areas, these units have far lower levels of staffing, programming and training than the CAPS units. We secured funding to convert four of these settings to the CAPS-level of services, which includes mental health treatment aides, dedicated social workers, psychologists and psychiatrists for each unit housing 18–25 patients. Thus far we have converted hospital return, step-up and diagnostic units to the CAPS-level of service, with promising results in clinical outcomes including medication adherence and exacerbations requiring hospital transfer. Each of these unit conversions costs approximately $1.5 million more than baseline commitments, almost exclusively in new staffing.

DOHMH and DOC are working to evolve the RHU units, but our clinical experience is that these units are very difficult to administer. The RHUs are designed to deliver punishment via solitary confinement at the same time that clinical staff are working to engage patients in group and individual therapy for 1–4 h per day. For many patients, the reward of moving from one hour out of cell to two hours out of cell is not a qualitative improvement. In addition, health and security staff on these units face very complicated tasks in getting the appropriate patients out of cell for the allotted times, leaving room for patients to not receive the time out of cell or other benefits they deserve and setting the stage for discord.

The data from both the CAPS and RHU units reveal that clinical improvements among incarcerated patients with mental illness are linked to less restrictive and more therapeutic approaches. These improvements are worthwhile but also quite costly. Each CAPS unit can house 18–25 patients and carries an annual cost of $1.5 million more than our pre-existing mental health units. While the staffing of these units is likely more costly than the solitary confinement units that they replace, these costs may be offset by decreased rates of injury, self-harm, use of force and ultimately, litigation. Our prior analysis revealed that for every 100 acts of self-harm, an additional 36 transfers to a higher level of care, 10 hospital admissions and 3760 hours escort time by security staff were incurred. This cost and increased likelihood that they are non-violent offenders also raises the important question about whether these patients might be appropriately connected to community clinical settings instead of incarceration. NYC has recently committed to several fundamental changes in how the criminal justice system interacts with persons with behavioral health concerns, including creation of diversion centers at the point of arrest and new screening (and potential diversion) in the arraignment settings [11]. These innovations are designed to identify and direct people some of those who currently go to jail with behavioral health concerns (including those with SMI) away from jail and towards community care. As these innovations are implemented in the coming year, our hope is to both broaden the reliance on clinical alternatives to solitary confinement inside the jail system, and reduce the number and share of persons with mental illness who enter the jails**.** Given the cost, recruitment and training requirements associated with creating CAPS style units, establishing robust community mechanisms to provide substance abuse, mental health treatment as well as supportive housing may result in lower costs and better clinical outcomes than creating large numbers these units inside jails.

## 5. Conclusions

Adverse health outcomes and basic human rights concerns support elimination of solitary confinement, particularly for adolescents and persons with mental illness. The CAPS design is a costly, but effective alternative to solitary confinement in jail settings. Rates of injury and self-harm appear to improve when patients are in treatment settings as opposed to solitary confinement. Costs of CAPS-style units may be offset by reductions in injury, hospitalization and litigation. However, it may be preferable to reduce the rates of incarceration for mentally ill persons altogether.
